# Identifying traditional Chinese medicine combinations for breast cancer treatment based on transcriptional regulation and chemical structure

**DOI:** 10.1186/s13020-025-01074-5

**Published:** 2025-02-14

**Authors:** Shensuo Li, Lijun Zhang, Wen Zhang, Hongyu Chen, Mei Hong, Jianhua Xia, Weidong Zhang, Xin Luan, Guangyong Zheng, Dong Lu

**Affiliations:** 1 Shanghai Frontiers Science Center of TCM Chemical Biology, Institute of Interdisciplinary Integrative Medicine Research, Shanghai University of Traditional Chinese Medicine, Shanghai, 201203 China; 2 School of Pharmacy, Second Military Medical University, Shanghai, 200433 China; 3 West China School of Public Health and West China Fourth Hospital, and State Key Laboratory of Biotherapy, Sichuan University, Chengdu, 610041 China

**Keywords:** Synergistic effect, Traditional Chinese medicine, Breast cancer, Transcriptional regulation, Chemical structure feature

## Abstract

Breast cancer (BC) is a prevalent form of cancer among women. Despite the emergence of numerous therapies over the past few decades, few have achieved the ideal therapeutic effect due to the heterogeneity of BC. Drug combination therapy is seen as a promising approach to cancer treatment. Traditional Chinese medicine (TCM), known for its multicomponent nature, has been validated for its anticancer properties, likely due to the synergy effect of the key components. However, identifying effective component combinations from TCM is challenging due to the vast combination possibilities and limited prior knowledge. This study aims to present a strategy for discovering synergistic compounds based on transcriptional regulation and chemical structure. First, BC-related gene sets were used to screen TCM-derived compound combinations guided by synergistic regulation. Then, machine learning models incorporating chemical structural features were established to identify potential compound combinations. Subsequently, the pair of honokiol and neochlorogenic acid was selected by integrating the results of compound combination screening. Finally, cell experiments were conducted to confirm the synergistic effect of the pair against BC. Overall, this study offers an integrated screening strategy to discover compound combinations of TCM against BC. The tumor cell suppression effect of the honokiol and neochlorogenic acid pair validated the effectiveness of the proposed strategy.

**Supplementary Information:**

The online version contains supplementary material available at 10.1186/s13020-025-01074-5.

## Background

The World Health Organization reports that cancer is the second leading cause of death worldwide, with an estimated 10 million deaths in 2020 [1]. Of all new female cancers, breast cancer (BC) accounts for about 30% each year and is the second leading cause of cancer-related death among women [2]. To combat this disease, various treatments, such as chemotherapy, hormonotherapy, and immunotherapy, have been developed to improve clinical outcomes in patients with BC [3]. However, challenges persist in achieving satisfactory effects due to the complex characteristics of the disease. For instance, BC exhibits significant molecular, pathological, and clinical heterogeneity. Molecularly, it can be categorized into four subtypes: luminal A, luminal B, human epidermal growth factor receptor 2-enriched, and triple-negative breast cancer. Drug resistance poses a significant challenge in BC treatment, particularly for advanced-stage cancers. Tamoxifen, an estrogen blocker, is a classic hormonotherapy that significantly reduces BC recurrence and mortality. However, 20–30% of tumors are resistant to tamoxifen therapy, presenting a fundamental limitation in clinical practice [4].

Drug combinations are widely recognized for their potential to improve treatment efficacy and overcome drug resistance when compared to single agents [5]. Tumors often develop diverse compensatory mechanisms that resist monotherapies. When a drug targets a specific pathway (e.g., estrogen receptor and human epidermal growth factor receptor 2 signaling), tumor cells may adapt by utilizing an alternative pathway to sustain their growth and survival [6]. For example, approximately 70% of BCs may develop resistance to hormonotherapy due to PI3K/AKT/mTOR pathway activation [7]. The strategic use of drug combinations targeting different pathways or mechanisms can enhance the likelihood of eradicating tumor cells and inhibiting the emergence of drug-resistant tumor cells. Furthermore, employing drug combinations permits the use of lower doses of each drug, thereby reducing the potential for harmful toxicity [8].

In recent years, numerous potential drug combinations for BC treatment have been proposed, including everolimus and exemestane, cetuximab and cisplatin, and docetaxel and doxorubicin [4, 5, 9]. However, exhaustively exploring the vast array of possible combinations remains a significant challenge, given the substantial investment of time and resources required. In silico methods, such as computer-aided drug discovery, offer promising advantages for exploring novel drug combinations due to their rapidity and efficiency [10]. For instance, Cheng and colleagues reported on the specific interaction mechanisms of effective drug combinations in treating diseases through protein network analysis [11]. Another study identified the key characteristics of the mechanism of action for synergistic cancer drugs [12]. Moreover, certain machine learning (ML) models, particularly deep learning (DL) models, have been developed to predict synergistic compound combinations for cancers based on publicly available high throughput screening datasets [13].

Extensive clinical experience spanning thousands of years has demonstrated the therapeutic effects of traditional Chinese medicine (TCM) in addressing health issues [14]. TCM, characterized by its use of herbal medicine and formulas containing various natural products, is known for its “multi-components, multi-targets, multi-activities” approach. The global recognition of TCM’s antitumor effects continues to grow through modern research [15], which primarily focuses on either whole formula or isolated individual compounds [16]. While studies on key components with synergistic effects could hold great promise for elucidating the advantages of TCM, identifying effective component combinations from its complex composition remains a significant challenge. The vast number of possible combinations makes experimental identification costly and time-consuming. Additionally, unlike approved or candidate small molecule drugs, only a few natural products with distinct targets or action mechanisms are suitable for combination prediction using in silico methods based on biological knowledge. Nevertheless, compound-perturbance transcriptome assays can aid in inferring a systematic influence at the gene or pathway level, thereby establishing a correlation between the compound and disease based on the principle of reversal effects [17]. However, the majority of these assays have focused on single-compound studies [18]. It is worth noting that gene sets, comprising closely related genes, can represent meaningful biological events such as biological processes and states, signaling pathways, and coexpressed modules, offering valuable insights for combination discovery. In addition, chemical structure features could also contribute to modeling the synergistic effects, which has been employed in many studies [19].

**Fig. 1:**
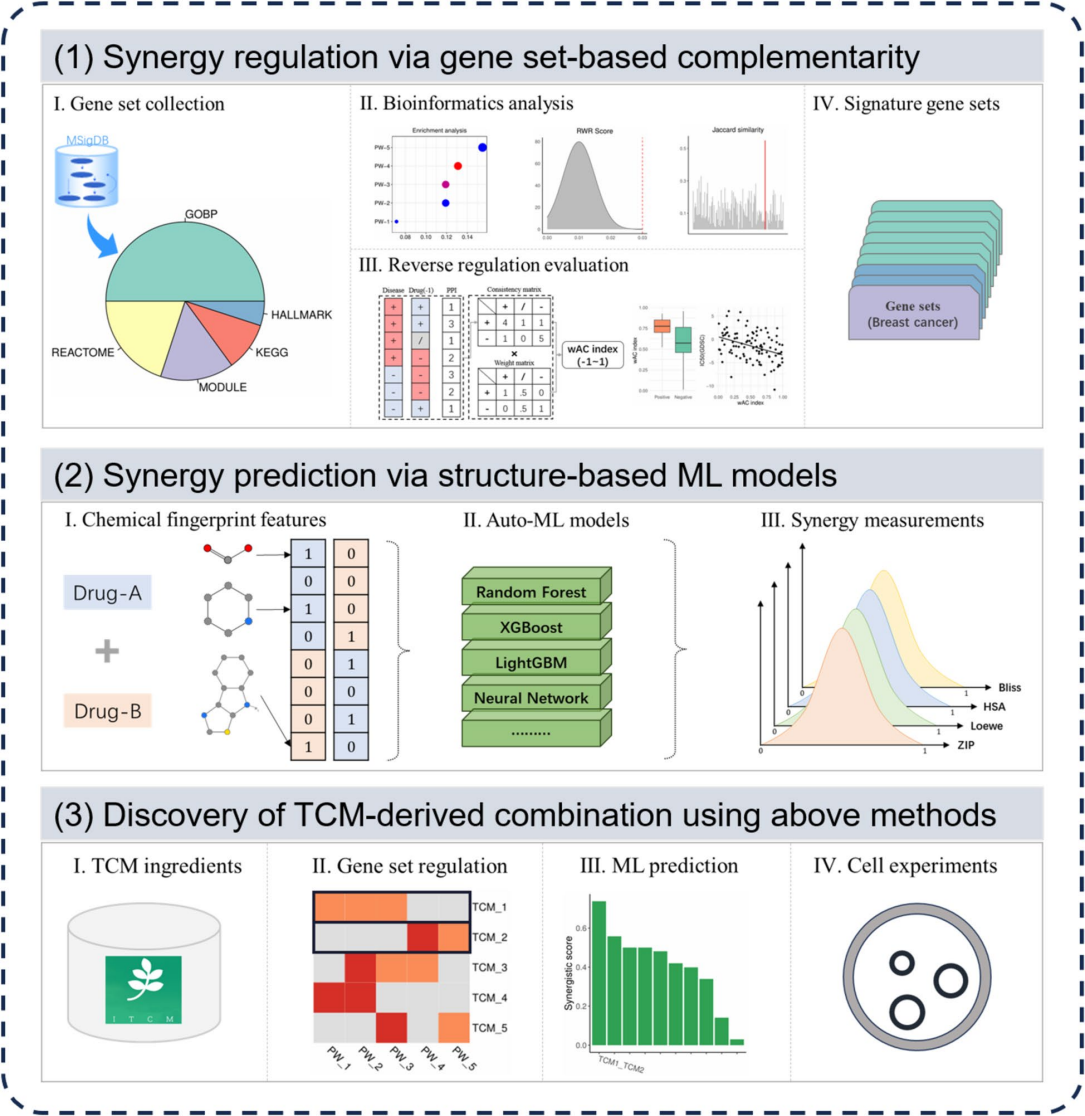
Discovery workflow of TCM-derived synergistic combinations against BC. Two in silico methods were applied to identify compound combinations with potential efficacy against BC. Effective combinations were identified based on synergistic effect of gene sets and high prediction scores from machine learning models, and finally confirmed through in vitro experiments.

Here, we developed an integrated computational approach to identify potential combinations of TCM natural products for the treatment of BC (Fig. 1). We collected thousands of gene sets representing various biological events to identify marker features associated with BC. These gene sets were then utilized to discover potential compound combinations with synergistic effect. In addition, we established machine learning models to predict synergy scores of compound combinations based on chemical structural features. Finally, we applied both methods to screen a large number of ingredients (*n* = 496) for the discovery of compound combinations. As a result, we identified the pair of honokiol and neochlorogenic acid (HONA) based on transcriptional regulation characteristics and high prediction scores, which was further confirmed through in vitro cell experiments.

## Materials and methods

### Collection of gene sets representing comprehensive biological events

We obtained 9940 gene sets associated with comprehensive biological events from the Molecular Signatures Database (https://www.gsea-msigdb.org/gsea/msigdb) [20]. Of these, we retrieved 7708 ontology gene sets and hallmark gene sets representing different biological processes or biological states. We then collected gene sets associated with signaling pathways in the Kyoto Encyclopedia of Genes and Genomes (*n* = 186) and Reactome (*n* = 1615) databases, two curated pathway databases based on evidence from the literature. We also collected 431 gene sets associated with cancer progression modules from pan-cancer studies.

### Identification of BC-related differentially expressed genes and dysregulated gene sets

The BC transcriptome dataset (1106 tumors and 113 normal samples) was downloaded from The Cancer Genome Atlas (TCGA) project through the TCGAbiolinks package of R software [21]. The expression count matrix of 19,934 protein-coding genes was extracted for differentially expressed gene (DEG) analysis via the DESeq2 pipeline [22]. The thresholds of BH adjusted *p*-value and absolute log2FoldChange were set to 0.01 and 1.0 to identify DEGs. Over-representation analysis (ORA) was performed to identify significant enrichment events correlated to BC based on these DEGs using the ClusterProfiler R package [23]. A gene set was defined as BC progression associated one when its constituent genes met the following criteria: (1) contained more than five and fewer than 500 genes and (2) had a significant enrichment score with adjusted *p*-values < 0.01.

### Distance calculation between gene sets and BC targets

The human protein-protein interactome (PPI) from a previous study [11] yielded a network containing 15,898 proteins (nodes) and 213,763 interactions (edges). Canonical targets associated with BC were retrieved from the TTD database [24]. The random walk with restart algorithm [25] was used to measure the proximity of each node to BC targets in the network. The distances of each gene set to these targets were calculated by aggregating the proximity of the constituent genes. The background distribution of each gene set was estimated by computing the proximity of 1,000 random permutations to targets. Adjusted *p*-values below 0.01 from the one-tailed test were considered significant.

### Redundancy evaluation between gene sets

We used the overlap coefficient (Eq. 1) to assess similarities between paired sets to eliminate redundancies of similar gene sets from different sources. Here, *P*_*A*_ and *P*_*B*_ represent sets A and B. The numerator represents the overlap between sets, and the denominator represents the smaller gene set. A coefficient of >0.5 indicates significant similarity. When two similar gene sets were identified, the one with higher significance in the enrichment analysis was retained for the subsequent study.

1 $$Overlap_{{\left( {P_{A} ,P_{B} } \right)}} = \left| {P_{A} \cap P_{B} } \right|/\min \left( {\left| {P_{A} } \right|,\left| {P_{B} } \right|} \right)$$

### Transcriptional profiles of the MCF7 cell line

Transcriptional profiles of compounds that perturb the growth of the MCF7 BC cell line were retrieved from the LINCS database (https://clue.io/data/CMap2020#LINCS2020). For any compound with multiple profiles, the following criteria were applied [26]: (1) the 24-h timepoint, and (2) the highest transcriptional activity score. Profiles of 2312 compounds recorded in the TTD database were retained, and 62 correlated with BC.

### Calculation of the wAC index

**Fig. 2:**
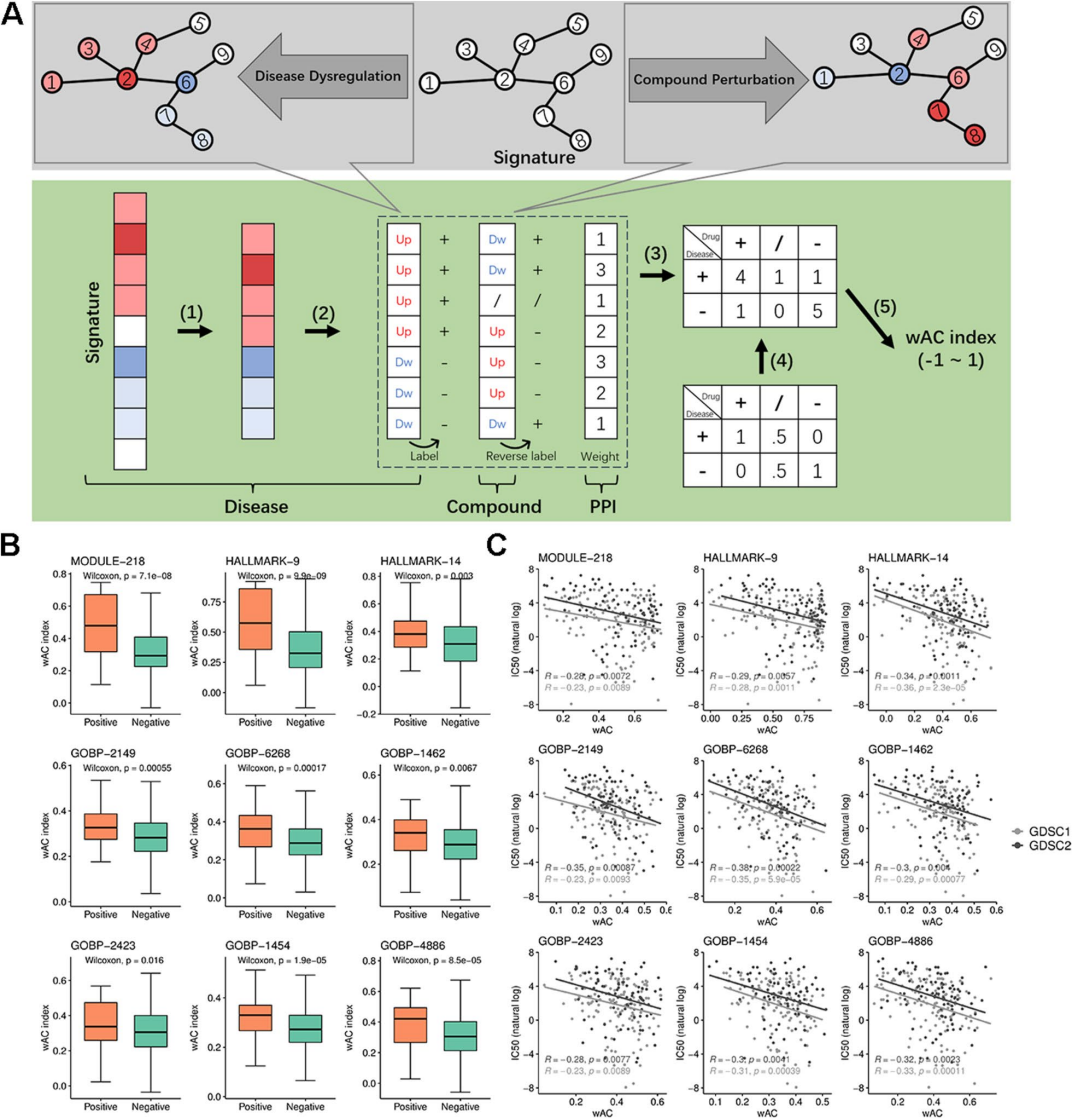
Recognition of signature gene sets based on the wAC index. **A** Schema of the wAC index calculation. For a specific gene set dysregulated in disease, the wAC index of one chemical perturbation can be calculated as follows: (1) extract DEGs of the dysregulated gene set; (2) label DEGs under disease conditions and chemical perturbation; (3) generate the consistency matrix; (4) design the corresponding weighted matrix; (5) and calculate the wAC index. **B** Box plots showing the significant differences of the wAC index on various gene sets between 62 BC drugs (Positive) and 2250 other drugs (Negative). **C** Scatter plots with fitted lines showing the correlation between the wAC index and natural log of IC_50_ in MCF7 cells of GDSC1 and GDSC2 drugs across signature gene sets. Significance was assessed by Pearson correlation analysis

The reversal effect was calculated as follows (Fig. 2A): first, BC up/downregulated (adjusted *p*-value < 0.01, absolute log2FoldChange > 0.5) genes were extracted and marked “+” or “−”. Expression profiles affected by the compounds were checked for reverse regulation. For the next step of the reverse consistency calculation, the opposite “−” or “+” labels with the same thresholds were assigned to up/downregulated genes in the treatment condition, and the residual genes were marked “0”. Thus, a label confusion matrix was generated to measure the reversal consistency between chemical perturbation and disease dysregulation at the gene set level. The AC1 (Agreement Coefficient 1) index was adopted by the observed and expected agreement proportions and implemented by the irrCAC R package in our study. This method is less biased for imbalanced categories (e.g., when most genes are upregulated in a gene set) compared to Cohen’s Kappa [27]. Additionally, we also considered importance of different genes within the set, based on their betweenness centrality in the PPI network. The consistency of genes with higher importance contributes more to the final index, resulting in the weighted AC1 (wAC). For each gene set, the wAC index ranges from −1 to 1, with higher values indicating stronger reverse consistency between breast cancer (BC) dysregulation and compound treatment.

### Measurement of the reversal effects of compounds against BC

According to the index, we identified signature gene sets for BC. First, the wAC index for a given gene set of BC drugs (positive group) was compared using Wilcoxon testing to non-BC drugs (negative group). We also collected MCF7 sensitivity (natural log of IC_50_) data from the Genomics of Drug Sensitivity in Cancer database (GDSC, https://www.cancerrxgene.org). Specifically, 129 drugs from GDSC1 and 89 drugs from GDSC2 were selected, as they overlapped with the LINCS drugs. We then calculated the correlation between the drug wAC index and sensitivity data for each gene set. If the wAC index of one set had higher values among BC drugs and negatively correlated to the natural log of IC_50_, which indicated a positive correlation with drug sensitivity, it was regarded as an essential gene set whose reverse regulation was thought to be associated with an underlying therapeutic role in BC. *p*-values < 0.01 were considered significant for all comparison and correlation analyses.

### Discovery of combination with synergistic regulation of gene sets

First, we gathered transcriptional profiles of 496 TCM-derived compounds on MCF7 cells from the ITCM database [28], and then the wAC index for each gene set was calculated. For each signature gene set ${P}_{i}$, the regulation score of a single compound ${D}_{1}$ is represented by ${S}_{{D}_{1},{P}_{i}}$. Based on the 50th and 80th quantiles of the wAC index among BC drugs, ${S}_{{D}_{1},{P}_{i}}$ of each single compound was graded as 0 (0–50%), 0.5 (50–80%), or 1 (80–100%), indicating weak, moderate, or strong effects for the gene set. For each compound ${D}_{1}$, we directly summed the grading scores (${S}_{{D}_{1},{P}_{i}}$) of the compound in all signature gene sets to represent the overall regulation score (denoted ${PS}_{{D}_{1}}$, Eq. 2, where *n* represents the number of signature gene sets). Typically, a single compound cannot comprehensively regulate all the signature gene sets. Therefore, we proposed another index (denoted ${TCS}_{{D}_{1},{D}_{2}}$, Eq. 3) to identify the potential combination (${D}_{1}, {D}_{2}$) with synergistic effect for more complete regulation. For each combination of two compounds, we first summed the regulation scores of the compounds on the same gene set ${P}_{i}$, capping the maximum value at 1. Then, the above scores of all signature gene sets were aggregated as TCS values. Finally, combinations with higher TCS values were selected for further screening.

2 $$PS_{{D_{1} }} = \mathop \sum \limits_{i = 1}^{n} S_{{D_{1} ,P_{i} }}$$

3 $$TCS_{{D_{1} ,D_{2} }} = \mathop \sum \limits_{i = 1}^{n} \min \left( {S_{{D_{1} ,P_{i} }} + S_{{D_{2} ,P_{i} }} , 1} \right)$$

### Drug combination data collection and ML modeling

Initially, four types of synergy scores (ZIP [zero interaction potency], Loewe, HSA, Bliss) [29] for 4966 unique combinations (involving 101 drugs) in the MCF7 BC cell line were obtained from the NCI-ALMANAC project and downloaded from SYNERGxDB (https://www.synergxdb.ca) [30]. Outliers for each type of synergy score were discarded based on the standard 1.5× interquartile range rule, and the remaining samples were subjected to min-max normalization. Three classic chemical fingerprint descriptors—MACCS (166 bits), CDK Substructure (307 bits), and PubChem (881 bits)—were selected based on the PaDELPy software [31] and each fingerprint of the two drugs was concatenated to represent the structural features of the combination. Each combination had two sample inputs, considering the concatenation order (e.g., Drug A–Drug B and Drug B–Drug A).

The overall data were firstly divided into training and test sets with an 80:20 ratio. Of note, combinations involving the same compounds but in different orders were consistently assigned to the same set. Subsequently, 9 machine learning regression models (ExtraTreesMSE, RandomForestMSE, XGBoost, CatBoost, LightGBMXT, LightGBM, LightGBMLarge, NeuralNetTorch, and NeuralNetFastAI) were constructed using fivefold cross-validation (CV) on the training set and evaluated on the test set using the Autogluon tool [32]. Multiple metrics, including root mean square error (RMSE), R squared (R^2^), mean absolute error, and median absolute error were computed. The optimal machine learning algorithm for each modeling context—defined by the input fingerprint and the output synergy metric—was selected based on the lowest RMSE value during cross-validation. For evaluation on the test set, the bagged predictions were averaged across the five models from each fold. When predicting the synergy scores for a TCM-derived combination, both concatenation orders of the fingerprints for the two compounds were utilized as inputs, and the average of these predictions was considered the final result.

### Cell cultures

The MCF7 human BC cell line was obtained from the Shanghai Institute of Biochemistry and Cell Biology, Chinese Academy of Sciences (SIBCB, CAS). The cells were cultured in 1640 medium (Gibco, 11875-093) with 1% penicillin–streptomycin (HyClone, SV30010) and 10% fetal bovine serum (Gibco, 10091148). Cultures were maintained at 37 °C (Thermo Fisher, USA) and 5% CO_2_.

### Cell proliferation assay and combination index

MCF7 cells were plated in 96-well plates (5000 cells/well), incubated overnight, and then treated with various concentrations of HO, NA, and HONA for 24 h. After treatment, 10 μL of CCK-8 solution (Meilunbio, China) was added, and the absorbance was measured at 450 nm using a BioTek Cytation 5 (Agilent Technologies, USA) after 2 h. Cell viability was calculated as follows: cell viability = [(AE − AB)/(AC − AB)] × 100%, where A is the absorbance, E is the experimental well, C is the control well, and B is the blank well. The Chou-Talalay method [33] was used to calculate the combination index (CI) to evaluate whether the combined effect of HONA is synergistic (CI < 1), additive (CI = 1), or antagonistic (CI > 1).

### Cell cycle and apoptosis assay

MCF7 cells were plated in 6-well plates at 2 × 10^5^ cells/well. After exposure to various concentrations of HO, NA, and HONA for 24 h, the cells were collected, washed with precooled PBS, and suspended in precooled 70% ethanol overnight at 4 °C. After removing the fixative, the cells were stained with 25 µL of propidium iodide, 10 µL of RNase A, and 500 µL of staining buffer (Cell Cycle Analysis Kit, Meilunbio, China). The samples were then incubated at 37 °C in the dark for 30 min and analyzed by flow cytometry (Beckman, USA).

Apoptosis assays were performed by collecting the treated cells, washing them with precooled PBS, and resuspending them in a binding buffer. Cells were stained with 5 μL of Annexin V-FITC and 10 μL of propidium iodide (Annexin V-FITC/PI Apoptosis Detection Kit, Meilunbio, China), gently mixed, incubated in the dark at room temperature for 10 min, and analyzed by flow cytometry.

### Reactive oxygen species measurement

The reactive oxygen species (ROS) assay kit (Beyotime, S0033S) was used to measure ROS levels after a 24-h exposure to HO, NA, and HONA. DCFH-DA was diluted in a serum-free culture medium to 10 µM, then added to the cells and incubated for 20 min in a 37 °C cell incubator. The cells were analyzed by flow cytometry.

### Colony formation assay

MCF7 cells were plated in 12-well plates (1.5 × 10^5^ cells/well) and allowed to adhere overnight. Varying concentrations of HO, NA, and HONA were added and incubated for 24 h. The cells were reseeded in 6-well plates (1000 cells/well) and incubated for two weeks, replacing the culture medium every 3 days. Colonies were stained with crystal violet and counted.

## Results

### Identifying 860 key gene sets related to BC

To gain a comprehensive understanding of the essential biological events of BC, we initially gathered 9940 gene sets (Supplementary Table 1). DEG analysis and ORA were conducted to identify critical genes related to BC by comparing expression profiles of tumor and normal samples. This led to the detection of 5015 significant DEGs, including 3018 upregulated and 1997 downregulated genes (Supplementary Table 2). Based on these DEGs, 860 gene sets were selected according to ORA enrichment scores (Supplementary Table 2). In summary, 15 gene sets defined biological states; 570 sets represented biological processes; 13 and 145 sets described signaling pathways of Kyoto Encyclopedia of Genes and Genomes and Reactome, respectively; and 117 sets depicted cancer modules.

### Acquiring 115 low-redundancy gene sets close to BC targets

The distance of gene sets to disease targets in the PPI network was evaluated to refine BC-related gene sets. One PPI network of over 15,000 proteins was constructed, and 69 were marked as BC targets according to the TTD database. For each gene set, the overall distance to disease targets was calculated using the random walk with restart algorithm. As a result, 318 of 860 gene sets were selected according to the distance permutation assay (Supplementary Table 3). We also discarded those sets with high similarity to others based on the overlap coefficient index, leaving 115 low-redundancy sets for an additional study (Supplementary Table 4).

### Inferring nine signature gene sets based on the wAC index

To identify signature gene sets associated with BC therapy, the wAC index was proposed to evaluate the transcriptional reversal effect of compounds based on gene sets (Fig. 2A). Two identification analyses were performed on compounds that generated substantial perturbation of transcriptomic data of LINCS project and other public datasets. We prioritized those sets that could be affected by BC drugs, showing higher wAC values than non-BC drugs (Fig. 2B). Gene sets with a negative correlation to log-transformed IC_50_ in MCF7 cells, indicating a significant association with drug sensitivity, were also investigated (Fig. 2C). These analyses yielded nine signature gene sets for BC treatment (Table 1, Supplementary Table 5). In summary, one set was related to the cancer module; two sets were associated with biological states, and the remainder belonged to different biological processes. Gene sets ranged from 89 to 479, and more than half of the genes were found to be aberrantly expressed in BC.

**Table 1:** The summary information for nine signature gene sets

ID	Type	Name	No. of genes	No. of DEGs	Overlap coefficient
MODULE-218	MODULE	MODULE 53	403	284	0.109
HALLMARK-9	HALLMARK	G2M CHECKPOINT	200	128	0.220
HALLMARK-14	HALLMARK	ESTROGEN RESPONSE LATE	200	137	0.070
GOBP-2149	GOBP	DEVELOPMENTAL MATURATION	294	169	0.082
GOBP-6268	GOBP	RESPONSE TO ALCOHOL	236	152	0.263
GOBP-1462	GOBP	RESPONSE TO METAL ION	350	199	0.206
GOBP-2423	GOBP	REGENERATION	188	109	0.165
GOBP-1454	GOBP	RESPONSE TO EXTRACELLULAR STIMULUS	479	253	0.150
GOBP-4886	GOBP	POSITIVE REGULATION OF CELL DIVISION	89	55	0.090

### Screening candidate TCM-derived combinations based on synergy regulation

**Fig. 3:**
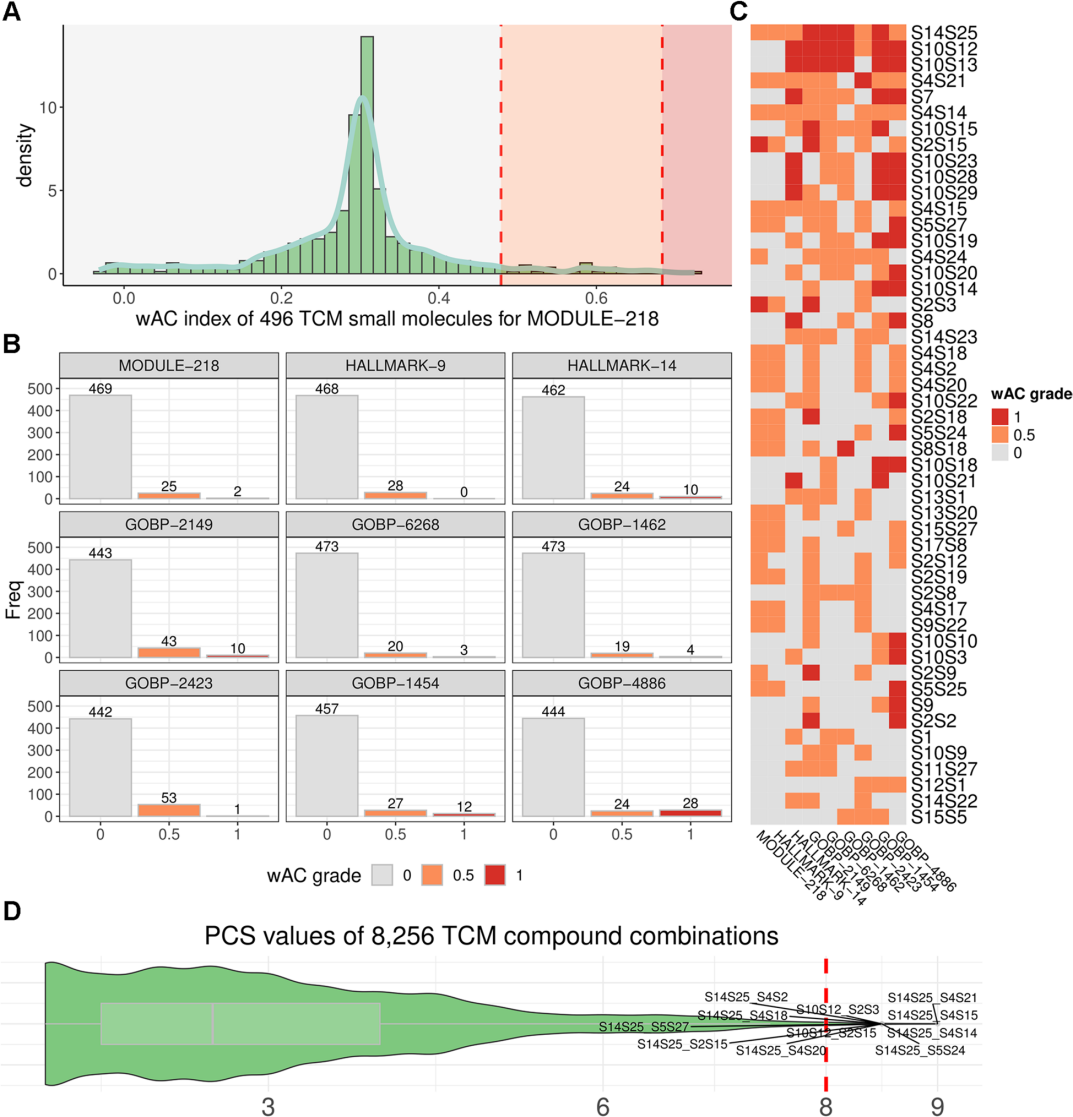
Regulatory effects of 496 TCM-derived components and their combinations on nine signature gene sets. **A** Density plots show the distribution of the wAC index of TCM compounds upon one gene set (MODULE-218), categorized into three grades based on the 50th and 80th percentiles of BC drugs. **B** Bar plots of the wAC-graded results of TCM compounds for each gene set. **C** Heatmap of the wAC-graded scores of the top 50 TCM compounds with the highest TCS values. **D** Violin plot of the TCS values of all 8256 TCM compound combinations. The *red dashed line* indicates the threshold of eight. Eleven combinations above the threshold are labeled

Combinations capable of exerting comprehensive transcriptomic regulation on key breast cancer signature gene sets are more likely to exhibit synergistic effects. To identify possible TCM compound combinations with synergistic effects against breast cancer, 496 natural products that lead to transcriptomic perturbations in MCF7 cells were first collected from the ITCM database. Then, the wAC indexes of these compounds on the nine signature gene sets were calculated. Regulation scores were graded based on the corresponding score distribution of BC drugs. For example, the 50th and 80th quantiles of the wAC index for BC drugs on MODULE-218 were 0.48 and 0.68, and thus three intervals (i.e., 0–0.48, 0.48–0.68, and 0.68–1.0) were used for grouping. As a result, 469, 25, and 2 compounds were classified as weak, moderate, and strong. The effect scores were recorded as 0, 0.5, and 1 (Fig. 3A).

The PS and TCS scores were introduced to evaluate the synergy regulation for each single compound and two-compound combination, respectively. As a result, 129 compounds showed a reversal effect (PS value > 0) on at least one gene set (Fig. 3B, Supplementary Table 6). The top 50 compounds with the highest PS values are presented in Fig. 3C. The S14S25 compound (Cinobufagin) showed a remarkable effect on all gene sets. We estimated the TCS values of 8256 possible combinations for these 129 compounds (Fig. 3D, Supplementary Table 7). Given a threshold greater than eight, 11 candidate combinations were identified, indicating significant regulation on all signature gene sets.

### Identifying potential combinations based on synergy prediction

**Fig. 4:**
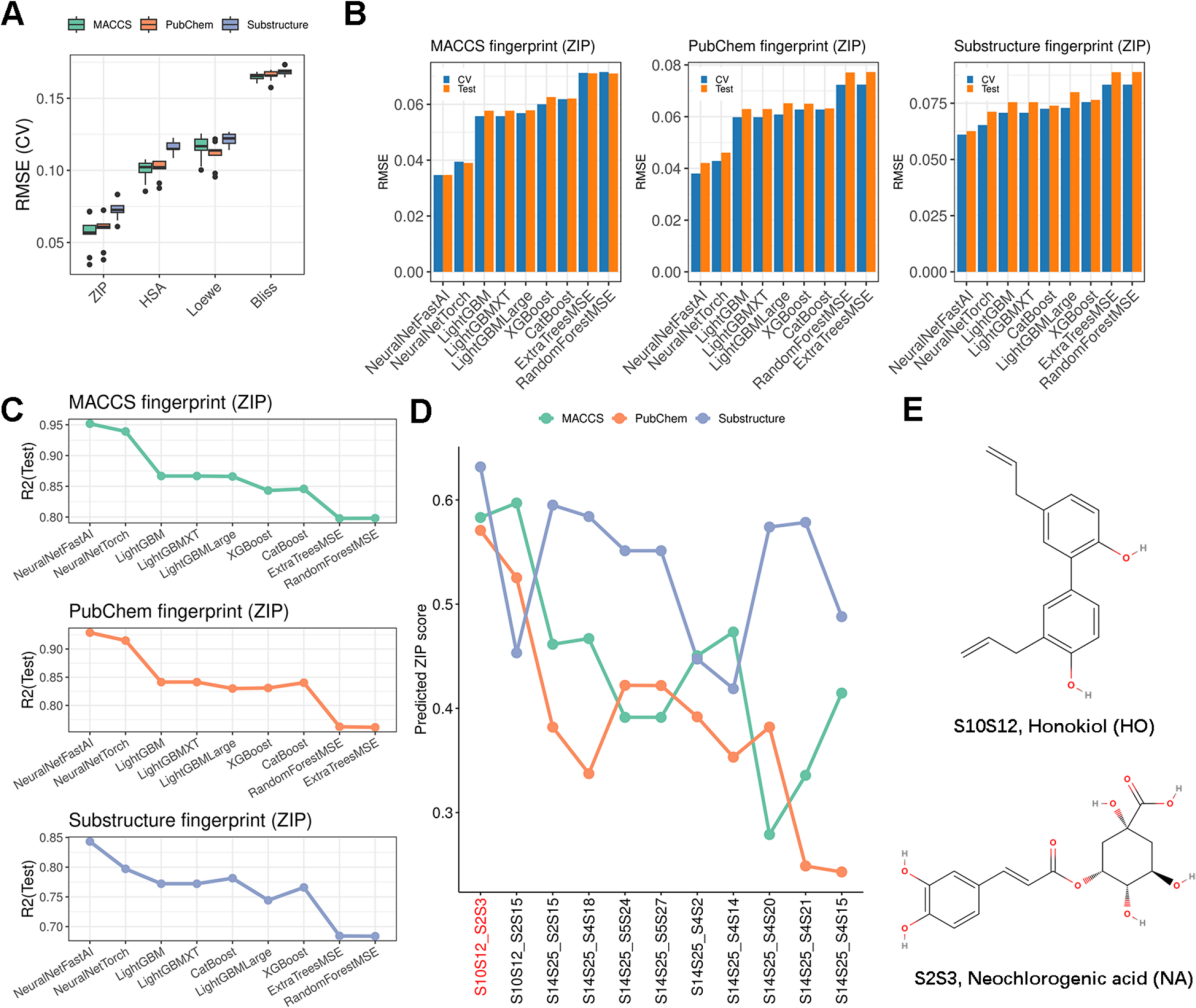
Machine learning modeling based on chemical structural features for synergy prediction. **A** Boxplots showing the RMSE distribution in the cross-validation set for nine ML algorithms in different model settings. Each model used one of three compound fingerprint types as the input and one of four synergy measurements as the output. **B** Bar plots show the RMSE values of nine machine learning algorithms with each of three fingerprint types as the input and ZIP synergy score as the output. *Blue bars* refer to cross-validation on the training set, and *yellow bars* refer to evaluation on the test set. *RMSE* root mean square error. *CV* cross-validation. **C** Line plots show the R^2^ values on the test set of nine machine-learning algorithms with each of three fingerprint types as the input and ZIP synergy score as the output. **D** Line plots show the predicted scores of 11 TCM-derived combinations using the best model of ZIP measurement with each of the three fingerprint types as the input. Combinations are sorted by the average score (the first one is marked in *red*). **E** 2D structures of Honokiol and Neochlorogenic acid

Chemical structure-based ML models were established to predict the synergy scores of compound combinations against MCF7 cells. Three chemical fingerprints (MACCS, PubChem, and Substructure) were used to train regression models for different synergy measurements (ZIP, Loewe, HSA, and Bliss), where one fingerprint was used as the input, and one synergy measurement was used as the output in each model setting. For each setting, nine ML algorithms were built through fivefold cross-validation on the training set and evaluated on the test set. We found that models of the ZIP measurement had lower RMSE values compared to other measurements, indicating the measurement might be more predictable according to the underlying chemical features captured by the fingerprints (Fig. 4A, Supplementary Table 8). The NeuralNetFastAI model demonstrated the lowest RMSE values for ZIP synergy measurement with each fingerprint as the input during cross-validation (Fig. 4B). Notably, the NeuralNetFastAI model for ZIP measurement with three different fingerprint types also exhibited excellent performance on the test set, as evidenced by the lowest RMSE and highest R^2^ values compared to other models (Fig. 4B, C).

In summary, the NeuralNetFastAI models for ZIP synergy using MACCS (CV RMSE: 0.035, Test RMSE: 0.035, Test R^2^: 0.95), PubChem (CV RMSE: 0.038, Test RMSE: 0.042, Test R^2^: 0.9), and Substructure (CV RMSE: 0.061, Test RMSE: 0.062, Test R^2^: 0.84) fingerprint types generally demonstrate the best performance. Besides, we furtherly prioritize features that contribute to modelling for each fingerprint type through permutation importance analysis (Supplementary Table 8). Therefore, we used these models to predict the ZIP scores for the 11 combinations screened by synergy regulation and identified that one pair, S10S12 (Honokiol, HO) and S2S3 (Neochlorogenic acid, NA), termed HONA, obtained the highest average predicted score (Fig. 4D, Supplementary Table 9), suggesting a potential synergistic effect of the two TCM-derived compounds (Fig. 4E). In addition, the DDI (Drug-Drug Interaction) prediction [34] to assess the potential toxicity of this combination, suggesting that HONA could exhibit a potentially favorable safety profile (Supplementary Table 10).

### Cell experiment verification of the synergistic effects of HONA

**Fig. 5:**
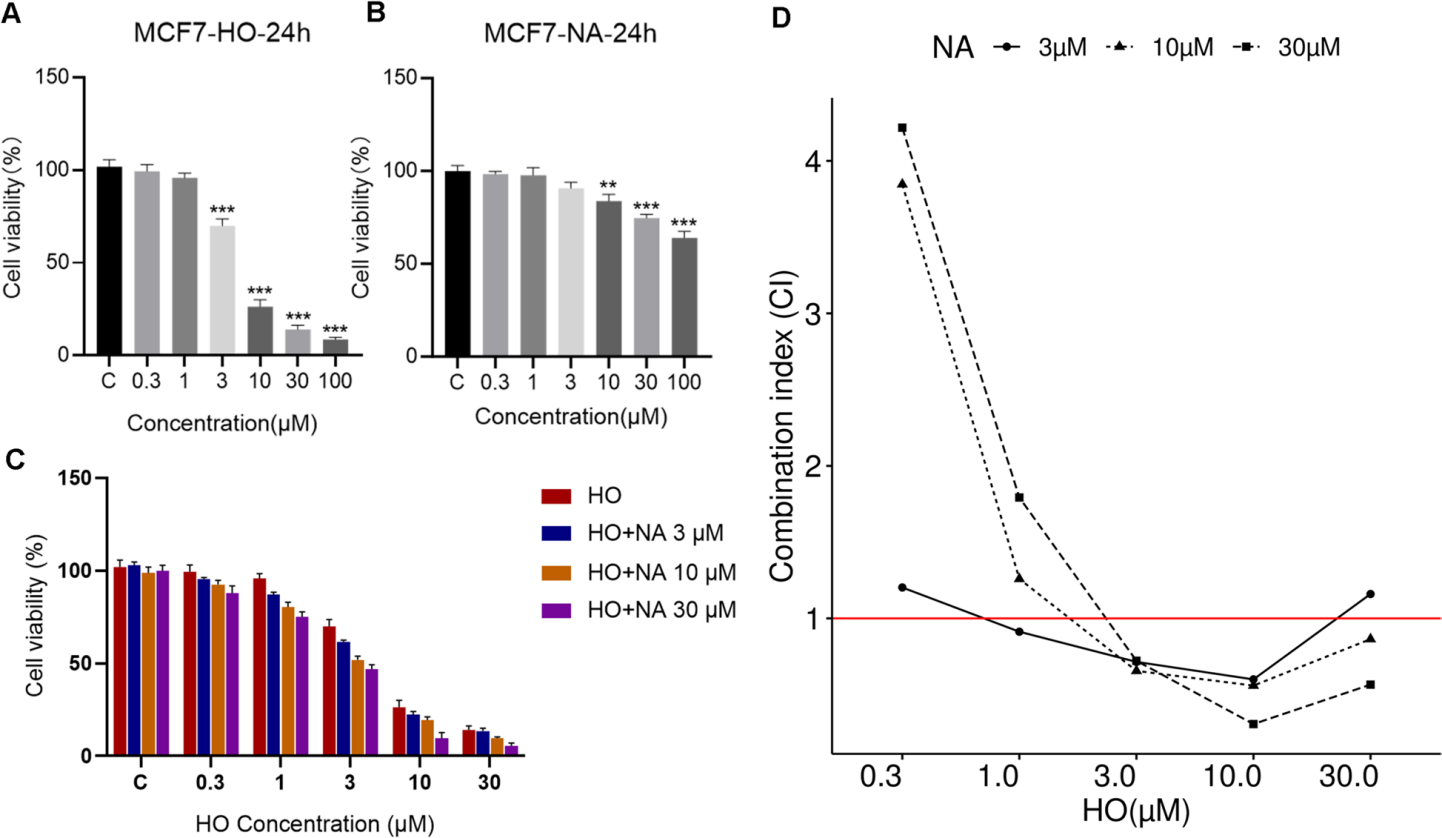
Effects of HO and NA on the proliferation of MCF7 cells. **A**, **B** MCF7 viability after treatment with different concentrations of HO or NA. C represents the untreated control group, where cells were cultured under standard conditions without exposure to HO or NA. Statistical analysis was performed using GraphPad Prism 7.0 software (La Jolla, CA, USA) and the statistical significance was evaluated by a two-tailed Student’s *t*-test. *p < 0.05, **p < 0.01, and ***p < 0.001. Data are shown as mean ± SD (n = 3). **C** MCF7 viability after treatment with different concentrations of HO or HONA. C represents the untreated control group. Data are shown as mean ± SD (n = 3). **D** CI values

To assess the combined impact of HO and NA (HONA), we initially studied the individual effects of varying concentrations of HO and NA on MCF7 cell viability. Our findings revealed a dose-dependent inhibition of MCF7 cell viability by both HO and NA, with HO showing particularly strong effects (Fig. 5A, B). Subsequently, we investigated their combined effects at different concentrations, observing a significant increase in cell viability compared to individual HO treatments (Fig. 5C). To quantitatively evaluate the optimal synergy of HONA, we calculated the CI for each concentration experiment. CI values for HONA were predominantly below 1 when the concentration of HO exceeded 1 μM, indicating a synergistic effect (Fig. 5D). Notably, the combination of 10 μM HO and 30 μM NA stood out, with a minimum CI of 0.306, demonstrating substantial synergy.

**Fig. 6:**
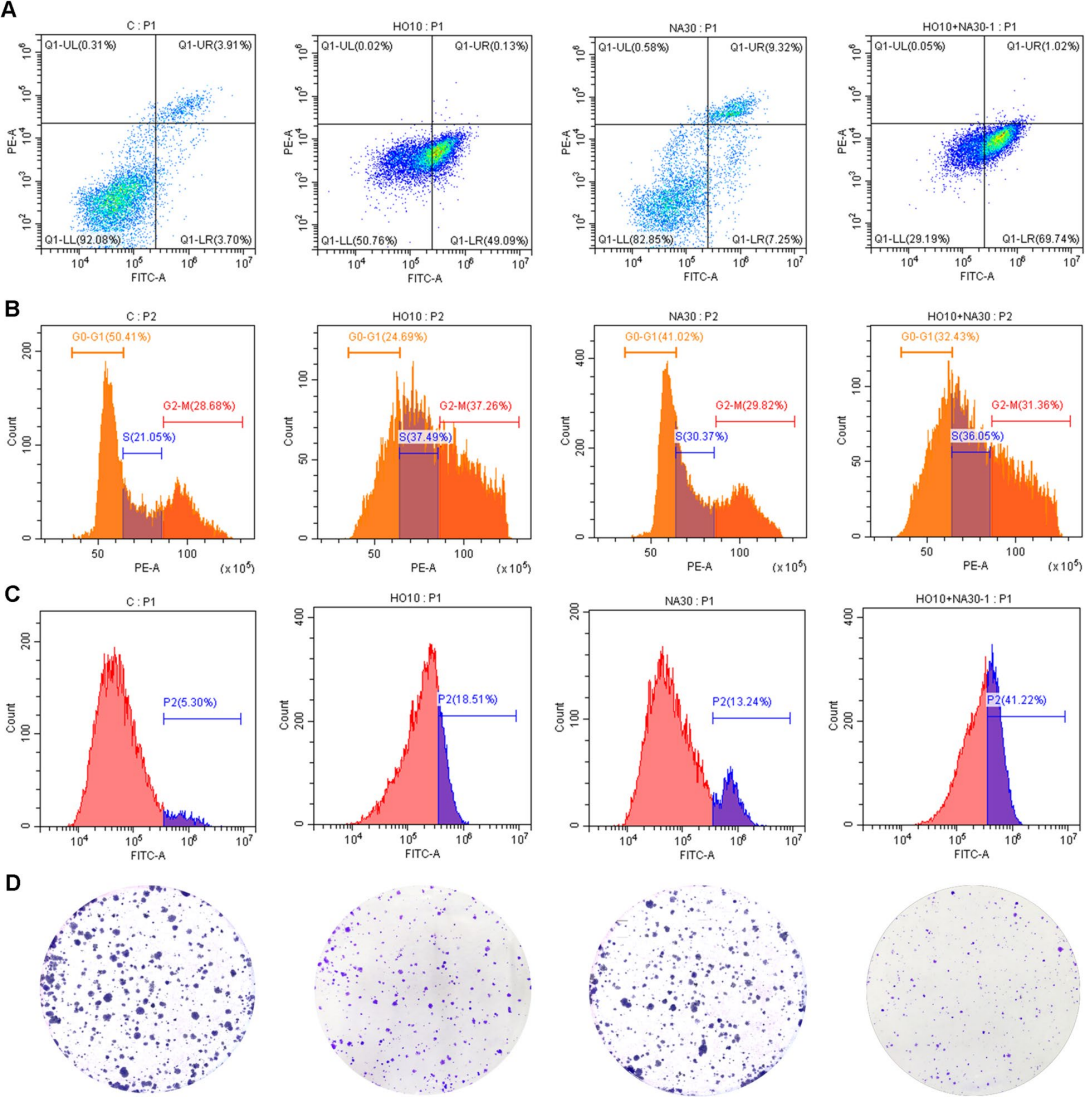
Synergistic investigation of HONA at concentrations with the lowest CI value in MCF7 cells in **A** cell apoptosis, **B** cell cycle, **C** ROS, and **D** colony formation assays. For each assay, the four subfigures from left to right represent the untreated control, 10 μM HO, 30 μM NA, and 10 μM HO plus 30 μM NA

A more detailed investigation of the combination at optimal concentrations was carried out using a cell apoptosis assay (Fig. 6A). The findings indicated a significant increase in early apoptosis, suggesting a stronger pro-apoptotic effect compared to each individual agent. In addition, the drug combination significantly arrested the S-phase of MCF7, as observed in the cell cycle assay (Fig. 6B). ROS levels were measured to assess the oxidative stress induced by the compound combination. There was a significant increase in ROS production following combination administration, indicating that the synergistic effect involves the generation of oxidative stress (Fig. 6C). Finally, the impact of the compound combination on the long-term survival and clonogenic potential of MCF7 was evaluated by colony formation assays. Consistent with the previous results, the combination produced a remarkable reduction in colony formation compared to each single agent (Fig. 6D).

## Discussion

BC, the most common malignant tumor in women, led to over 685,000 reported deaths and an estimated 2.3 million new cases in 2020 [1]. Over the years, various treatment options such as surgery, chemotherapy, radiation therapy, hormone therapy, targeted therapy, and immunotherapy have collectively contributed to improving survival. However, due to the significant heterogeneity in disease pathology, genomic alterations, gene expression, and the tumor microenvironment, BC exhibits resistance to many therapies [35]. Combining drugs has gained attention as it could make it challenging for tumor cells to develop resistance to multiple drugs with synergistic action. While previous computational models for combination discovery are most based on distinct targets or mechanism of action, they have been relatively limited for natural products of TCM [11, 12, 36]. Given the extensive history and multicomponent nature of TCM, it presents a complex landscape for discovering effective combination therapies. Meanwhile, synergistic combinations are more effective in explaining its therapeutic effects on breast cancer and other diseases compared to individual compounds. Here, our study integrates bioinformatics and machine learning to systematically uncover potential synergistic TCM-derived compounds for BC treatment. Using omics data, we applied the hypothesis of transcriptional regulation across signature gene sets to identify synergistic combinations. With the aid of cheminformatics, we then encoded compound structures and leverage high-throughput screening data to build machine learning models targeting BC-specific synergy. This integrative computational strategy not only provides a more systematic and data-driven method for discovering compound combinations compared to traditional trial-and-error ways in clinical settings, but also offer new insights for the research of compound-based Chinese medicine [37].

The identification of gene sets that have a compound combination with synergistic regulation on multiple BC-related biological events could be crucial in overcoming resistance and reducing side effects for better therapeutic outcomes. In our study, we initially identified 860 gene sets based on DEGs and ORA in TCGA-Breast Invasive Carcinoma cohorts. We then narrowed it down to 115 low-redundancy sets closely linked to BC targets. We introduced the wAC index to infer the potential association between drugs and diseases by evaluating the reverse consistency of transcriptional regulation upon gene sets. This led us to pinpoint nine signature gene sets whose reverse regulation was specific to BC drugs compared with non-BC drugs and significantly correlated with drug sensitivity on MCF7 cells. Some of these gene sets are known to be closely associated with BC, such as HALLMARK-9 and GOBP-4886, which are involved in regulating cell proliferation, a critical factor in developing and treating many tumors [38]. In addition, HALLMARK-14 is associated with estrogen signaling, which plays a role in the progression of BC, as the majority of human BCs initiate as estrogen-dependent [39]. The GOBP-6268 process focuses on the response to alcohol, and recent research shows that alcohol has a complex impact on BC development, including disruption of the extracellular matrix and promotion of epithelial-mesenchymal transition [40, 41]. Lastly, GOBP-1462 highlights the role of metal ions in crucial biological processes, including cell signaling, DNA synthesis and repair, and redox reactions [42].

Machine learning models were trained to predict the synergy of compound combinations in MCF7 BC cells based on their chemical structural features. Four synergy measurements (ZIP, Loewe, HSA, and Bliss) were considered, and it was observed that the models based on the ZIP measurement generally exhibited satisfactory performance. This suggests that the machine learning models for the ZIP measurement were more effective at learning from the structural features of compound combinations. The ZIP synergy metric was highlighted for its ability to capture drug interaction relationships by comparing changes in the potency of dose-response curves between individual drugs and their combinations [43]. Three common fingerprint types—MACCS, PubChem, and Substructure—were used to encode substructure and pattern features from different perspectives [44]. The best machine-learning model built on each fingerprint within the Autogluon framework was employed to predict the ZIP synergy scores of the screened combinations from the previous step. Besides, we performed the permutation importance analysis to assess the relative significance of features across different fingerprint types, which could optimize future combination designs.

Thereafter, we combined transcriptional regulation and structure-based prediction models to investigate the synergy of 496 TCM compounds against BC. First, we postulated that compounds capable of significantly perturbing the expression of BC-related signature gene sets may exhibit synergistic regulatory effects that disrupt critical cancer pathways. Second, we hypothesized that compounds with specific chemical structure features may enhance or potentiate each other's therapeutic effects. For the former method, 129 compounds with non-zero PS values on signature gene sets were identified for subsequent screening. Notably, S14S25 (Cinobufagin) demonstrated a significant effect on all signature gene sets, ranking first among the 129 TCM compounds. This active natural product is derived from the dried secretion of the postauricular gland or skin gland of *Bufo gargarizans* Cantor or *Bufo melanostictus* Schneider, common in Chinese medicine [45, 46]. Recent studies have highlighted its potential therapeutic role in BC [47], validating the rationale behind the screening methods based on gene sets. Subsequently, we screened 11 candidate combinations based on the TCS evaluation, all of which exhibited TCS values above 8, indicating a significant reversal effect on all signature gene sets. Finally, based on the ZIP synergy scores predicted by ML models, the combination of S10S12 (HO) and S2S3 (NA), termed HONA, was identified as a promising compound pair for BC treatment.

Initially, we evaluated the potential toxicity of HONA by the Way2Drug tool and the results suggested the combination could have low probabilities of common adverse effect and weak interaction mediated by P450. HO, a lignan compound derived from Magnolia species such as *Magnolia grandiflora* and *Magnolia dealbata*, demonstrates pleiotropic effects, particularly antitumor bioactivity [48, 49] and low toxicity in many in vitro and in vivo studies [50] This compound can reversely regulate over half of the biological events associated with BC, including GOBP-4886, GOBP-1454, GOBP-2423, GOBP-1462, GOBP-6268, GOBP-2149, HALLMARK-14. These pathways are central to BC progression, as they influence hormone response, cellular repair, stress adaptation, and proliferation. NA is an isomer of chlorogenic acid and can be found in various natural plants, including honeysuckle. It has been reported to possess anti‑inflammatory and antitumor properties [51, 52] and show safety in vitro [53, 54]. In our transcriptomic analysis (Fig. 3C), NA could act on MODULE-218 and HALLMARK-9 compared with HO, indicating an essential role in DNA integrity and damage checkpoint signaling before mitosis. This could be the potential synergistic mechanism underlying the improved inhibition of BC cells, meaning that HO and NA could synergistically impair tumor growth by targeting distinct, yet interconnected, oncogenic processes. Although the machine learning models used in this study cannot explicitly attribute synergy to specific structural fragments due to their black-box nature, feature importance analyses were conducted from three fingerprints (MACCS, PubChem, and CDK substructures) to identify the substructure features that contribute most to the model predictions.

Finally, in vitro experiments for HONA confirmed the dose-dependent responses to each individual compound and the synergistic effect of the compound pair. HO demonstrated a potent dose-dependent inhibitory effect, while NA also showed significant inhibition, albeit to a lesser extent. Importantly, their combination (HONA) consistently outperformed individual treatments in reducing cell viability, particularly at higher concentrations of HO. To quantitatively assess the synergistic effects of different dose combinations, we calculated combination index (CI) values using the Chou-Talalay method. Among all tested combinations, 10 μM HO combined with 30 μM NA yielded the lowest CI value (0.306), indicating the strongest synergy. Based on the optimal concentration combination, additional assays on cell apoptosis, cell cycle, ROS levels, and colony formation provide further in vitro validation of the synergistic effect of HONA against BC.

## Conclusions

In summary, our research represents a significant step forward in understanding the combined potential of TCM compounds for BC treatment. The HONA combination validated by experimental assays serves as a promising example of the effectiveness of our integrated computational approach. Moving forward, further pharmacological investigations, including animal experiments and toxicity assessments, will be essential to fully confirm the synergistic benefits and ensure the safety of these findings for their potential application in the treatment of breast cancer. Finally, our study’s integration of expression-based regulation synergy and structure-based machine learning model prediction presents an innovative method for identifying combinations of TCM natural products for BC, with the potential for extension to other common cancers in the future.

## Supplementary Information

Additional file 1. Supplementary Table 1.

Additional file 2. Supplementary Table 2.

Additional file 3. Supplementary Table 3.

Additional file 4. Supplementary Table 4

Additional file 5. Supplementary Table 5

Additional file 6. Supplementary Table 6

Additional file 7. Supplementary Table 7

Additional file 8. Supplementary Table 8

Additional file 9. Supplementary Table 9

Additional file 10. Supplementary Table 10

## Data Availability

The essential analysis code, together with the summary diagram and public datasets source are available at GitHub (https://github.com/lishensuo/tcm_mol_comb). Other data will be made available on request, please contact the corresponding author.

## Abbreviations

BC: Breast cancer
CI: Combination index
DEG: Differentially expressed gene
DCFH-DA: 2′-7′-Dichlorodihydrofluorescein diacetate
HO: Honokiol
NA: Neochlorogenic acid
ORA: Over-representation analysis
PPI: Protein–protein interactome
RMSE: Root mean square error
ROS: Reactive oxygen species
TCGA: The Cancer Genome Atlas
TCM: Traditional Chinese medicine
